# Emotion perception through the nose: how olfactory emotional cues modulate the perception of neutral facial expressions in affective disorders

**DOI:** 10.1038/s41398-024-03038-z

**Published:** 2024-08-24

**Authors:** Elisa Dal Bò, Cinzia Cecchetto, Alejandro Luis Callara, Alberto Greco, Francesca Mura, Nicola Vanello, Fabio Di Francesco, Enzo Pasquale Scilingo, Claudio Gentili

**Affiliations:** 1 Department of General Psychology, University of Padova, Padua, Italy; 2 Department of Information Engineering, University of Pisa, Pisa, Italy; 3 Research Center “E. Piaggio”, University of Pisa, Pisa, Italy; 4 Department of Chemistry and Industrial Chemistry, University of Pisa, Pisa, Italy

**Keywords:** Human behaviour, Neuroscience, Psychiatric disorders

## Abstract

Humans can decode emotional states from the body odors of the conspecifics and this type of emotional communication is particularly relevant in conditions in which social interactions are impaired, as in depression and social anxiety. The present study aimed to explore how body odors collected in happiness and fearful conditions modulate the subjective ratings, the psychophysiological response and the neural processing of neutral faces in individuals with depressive symptoms, social anxiety symptoms, and healthy controls (*N* = 22 per group). To this aim, electrocardiogram (ECG) and HD-EEG were recorded continuously. Heart Rate Variability (HRV) was extracted from the ECG as a measure of vagal tone, event-related potentials (ERPs) and event-related spectral perturbations (ERPSs) were extracted from the EEG. The results revealed that the HRV increased during the fear and happiness body odors conditions compared to clean air, but no group differences emerged. For ERPs data, repeated measure ANOVA did not show any significant effects. However, the ERPSs analyses revealed a late increase in delta power and a reduced beta power both at an early and a late stage of stimulus processing in response to the neutral faces presented with the emotional body odors, regardless of the presence of depressive or social anxiety symptoms. The current research offers new insights, demonstrating that emotional chemosignals serve as potent environmental cues. This represents a substantial advancement in comprehending the impact of emotional chemosignals in both individuals with and without affective disorders.

## Introduction

Depression and social anxiety are among the most impairing psychiatric disorders and are both characterized by social impairment and dysregulated affective and motivational disposition. Social anxiety presents intense fear and avoidance of social situations [1, 2], while depression presents a lack of motivation for social interaction, fostering feelings of loneliness, disconnection, and social rejection [3]. Importantly, social functioning difficulties are present not just among those diagnosed with major depressive disorder, but also among individuals experiencing subclinical depressive symptoms [4, 5]. In addition, social isolation and feelings of loneliness have been linked to adverse health outcomes, such as poorer cardiovascular function and reduced sleep quality, as well as a deterioration of mental health [6–9].

In the context of psychological disorders such as depression and social anxiety, dysregulation of the motivation to approach rewards and avoid threats plays a significant role in the development and maintenance of the disorders [3, 10–12]. Approach and avoidance motivational systems are integral components of human behavior, influencing individuals’ responses to stimuli and guiding decision-making processes [13–15]. Approach motivation involves the pursuit of positive outcomes, rewards, or goals, driven by the anticipation of pleasure and satisfaction. In contrast, avoidance motivation is described as the desire to avoid negative outcomes, threats, or punishments. Individuals are motivated to refrain from unpleasant or aversive stimuli, leading to behaviors aimed at minimizing or escaping potential harm. In this regard, depression is characterized by a blunted approach motivation, resulting in anhedonia, apathy, and psychomotor retardation, and leading to reduced interest and motivation in pursuing rewarding experiences or goals. On the other side, individuals with social anxiety exhibit an overactive avoidance motivation, driven by fear of social evaluation and rejection, resulting in avoidance of social interactions and situations. Both conditions are considered highly impairing, ranking among the top five psychiatric disorders in terms of social dysfunction [16].

The study of appetitive and affective motivation has traditionally emphasized acoustic and visual aspects of human social interaction, by analyzing the emotional response to pleasant or unpleasant images, used to elicit the activation of the appetitive and defensive motivational systems. There is consistent evidence reporting reduced processing of pleasant pictures (compared to negative and neutral ones) in individuals with clinical and subclinical depression, measured with electroencephalographic (EEG) event-related potentials (ERPs) [17–19], EEG frequency bands [20–22], or startle eyeblink reflex [23–25], but also reduced activation for reward anticipation and receipt [26, 27]. On the other side, individuals with social anxiety often display heightened attention toward fear-related emotional faces during the initial stages of processing [28–31]. This tendency aligns with their increased sensitivity to threatening stimuli, characteristic of social anxiety.

A growing number of evidence suggests that humans can decode emotional information also from the body odors of conspecifics (also called chemosignals) [32–35]. Indeed, it has been shown that exposure to a body odor produced during an emotional experience can trigger, in those perceiving that odor, the partial reproduction of emotion-like responses [36], a phenomenon called “emotional contagion” [37]. The majority of evidence focused on negative emotions, such as fear or stress. Body odors produced during such emotional states can induce in perceivers a state of vigilance [38, 39] and facilitate subliminal perception of fearful facial expressions [40, 41]. Research has demonstrated that fear body odor can also affect the facial muscle activity of recipients, notably by heightening the activity of the medial frontalis muscle, which is associated with the expression of fear on the face [42–44]. In addition, growing evidence suggests that positive emotions, such as happiness, can also be communicated through body odors. As evidence suggests, happiness body odors can induce facial expressions of happiness [45], increase creativity and reduce heart rate in receivers [46, 47]. Interestingly, the decoding of olfactory information can occur even when the body odor is not consciously perceived, such as when masked by fragranced products [48–50] or when presented in very low concentrations [33, 40, 51], making them an effective type of contextual cues [38, 50].

The ability of body odors to convey emotional information is particularly relevant in all conditions in which social interaction and emotional processing are impaired, as in affective disorders. Given that the processing of human chemosignals has been shown to be largely unaffected by the allocation of attentional resources [33] the use of positive and negative emotional chemosignals could shed light on the complex interplay between the two motivational systems in the development and course of affective disorders. However, to date, only a few studies investigated the perception of chemosignals in affective disorders. Social anxious individuals reported enhanced startle reactivity to [52] and faster processing of anxiety chemosensory signals compared to healthy controls [53], an effect similar to that obtained with threatening visual stimuli [54, 55]. Moreover, when visual (fearful faces) and olfactory (chemosensory anxiety signals) stimuli were presented together, highly social anxious individuals showed larger withdrawal-related motor behavior and enhanced neuronal processing compared to non-social anxious individuals [56]. In addition, when investigating the olfactory metacognitive abilities, individuals with social anxiety reported reduced awareness of chemosensory signals [57]. Conversely, recent research indicates that individuals with depressive symptoms report increased awareness of chemosensory signals [57] and that stress-related chemosignals enhance perspective-taking and affective responsiveness to grief in individuals with depression [58].

The current study was designed to investigate, for the first time, social motivation toward both negative (fear) and positive (happiness) emotional chemosignals in individuals with symptoms of depression (DEP) and with symptoms of social anxiety (SAD), extending beyond previous research by encompassing a broader range of emotional stimuli and target populations. The study adopts a comprehensive methodology by examining subjective, peripheral, and neural responses to neutral facial expressions presented alongside emotional chemosignals. This multifaceted approach offers a more nuanced understanding of how emotional chemosignals impact both subjective experiences and underlying psychophysiological processes. Electroencephalography (EEG) was used to measure cortical dynamics, employing both the event-related potential (ERP) analysis and the time-frequency analysis of the EEG activity within specific frequency bands to provide a simultaneous examination of affective disposition and cognitive processing. In addition, emotional reactions to chemosignals were collected both directly, through self-report measures of arousal and valence indices, and indirectly, through heart rate variability (HRV). In the depression group, given the blunted response toward positive stimuli, reflected by a hypoactivation of the appetitive motivational system, we expected that the unconscious presentation of the happiness chemosignal would enhance the activation of the appetitive motivational system, leading to increased processing of neutral faces compared to a clean air condition. Conversely, the social anxiety group was expected to show heightened processing and perceived arousal toward neutral faces during the fear condition compared to clean air, reflecting the hyperactivation of the defensive motivation system that characterizes individuals with social anxiety.

## Materials and methods

### Participants

The study was conducted with the adequate understanding and written consent of the participants in accordance with the Declaration of Helsinki and was approved by the local Ethics Committee, University of Padua (prot. no. 3667). Informed consent was obtained from all participants. The study was preregistered at the Open Science Framework (https://osf.io/ayte9). Only female participants were included and were pre-screened to ensure eligibility. In order to be included in the study, participants were pre-screened with online questionnaires to exclude the presence of: chronic rhinitis or other conditions that may affect the ability to perceive odors, smoking, pregnancy or breastfeeding, presence of other mental disorders (including substance abuse disorders) apart from Major Depression, Chronic Depression, Minor Depression, or Dysphoria and Social Anxiety Disorder, presence of any severe somatic or neurological conditions, use of psychotropic drugs at the moment of the recruitment (including antidepressants, antipsychotics, anxiolytics, and mood stabilizers), presently undergoing psychological therapy, presence of severe psychotic symptoms (i.e., hallucinations and/or delusions), presence of suicidal thoughts, incapability to understand and to give informed consent for the experiment, being younger than 18 years or older than 35 years, being left-handed, no previous diagnosis of COVID-19. Moreover, participants were recruited if they could be included in one of the three experimental groups: individuals with symptoms of depression (DEP), individuals with symptoms of social anxiety (SAD) or healthy controls (CONT). Inclusion criteria were a score over or equal to 50 on the Liebowitz Social Anxiety Scale in its self-report formulation (LSAS-SR) for the SAD group, a score over or equal to 5 on the Patient Health Questionnaire (PHQ-9) for the DEP group, and a score below 5 on PHQ-9 and an LSAS score below 40 for the CONT group. Those subjects fulfilling the criteria were invited for a lab clinical interview, during which the presence or absence of the disorder was confirmed with the Structured Clinical Interview for DSM-5 (SCID-5-CV; modules A and F). Moreover, participants were screened for normative olfactory function with the Sniffin’ Sticks test (Burghart Instruments, Wedel, Germany [59]; see Supplementary material for a description of the test), and only normosmic participants were included.

Our target sample size was 75 participants divided into three groups (DEP, SAD, CONT). The sample size was evaluated using simulations obtained with *ANOVA_exact* analysis [60] based on specific hypotheses about changes across groups and conditions. The effect size was based on an analysis of literature results when available [56, 61]. Specifically, the change in LPP mean level observed in the happiness chemosignal condition with respect to clean air for the depressed group was supposed to be equal to 50% of the LPP standard deviation. Moreover, the change in the mean level of LPP in the social anxiety group during the fear chemosignal condition with respect to the clean air condition was supposed to be equal to 80% of the LPP standard deviation. The correlation among within-subject measures was equal to 0.5. The partial eta [2] for group-chemosignal interaction was 0.08 and the achieved power was 50% of the LPP standard deviation of 0.8 with 25 subjects per group. The study initially enrolled 79 participants, but data from 13 were excluded due to technical problems, resulting in a final sample of 66 participants (22 CONT, 22 DEP, 22 SAD), each receiving €25 for participation. Data from seventeen participants included in the CONT group were part of another EEG analysis published in [62].

### Stimuli

For the passive viewing task, 126 neutral faces (63 females, 63 males) were selected from the Chicago face database [63]. The images were distributed over the 21 blocks constituting the task in order to match for physical facial features, age of the actors, attractiveness, femininity, masculinity, trustworthiness and for the seven levels of emotional expressiveness (ratings from [63]). Moreover, six faces were used for training purposes. All faces were presented on a white background.

Three odor conditions were presented during the experimental task: clean air, fear chemosignal, happiness chemosignal. Chemosensory signals were collected from a separate group of healthy individuals exposed to video clips inducing happiness or fear, and “super-donors” were created to reduce interindividual variability in the collected sweat samples. Details of sweat sample collection and presentation are outlined in the Supplementary material. Odors were delivered with a custom-built, continuous airflow, computer-controlled olfactometer with 3 lines: one providing odorless air and the other two connected to the airtight jars containing the super-donor pads (fear and happiness). Odorous or odorless air was delivered directly to both nostrils with a nasal cannula, with constant airflow kept between 50 and 70 ml/min.

### Physiological recordings

EEG was recorded continuously using a 256-channel Geodesics EGI System (Electrical Geodesics, Inc., Eugene, Oregon, USA) with a sponge-based Geodesic Sensor Net. The sensor net was aligned with respect to four anatomical landmarks: two pre-auricular points, the nasion and the inion. Electrode-to-skin impedances were kept below 50 kΩ. The sampling rate was 500 Hz and electrode Cz was used as the reference.

The electrocardiogram (ECG) was recorded continuously using Ag/AgCl surface electrodes that were positioned on the participant’s chest in a modified lead II configuration. Electrodes were connected to a wearable ECG device (Shimmer3 ECG Unit, Shimmer 2018, Realtime Technologies Ltd, Dublin, Ireland), worn on the chest with an elastic band, connected to Consensys v1.6.0 software for recording via Bluetooth.

### Procedure

**Fig. 1:**
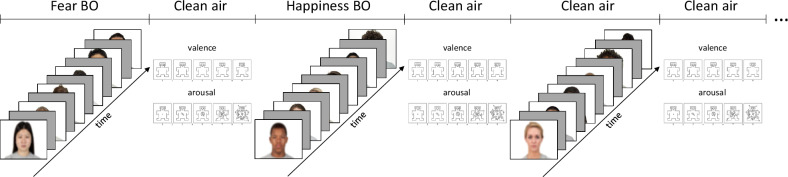
Experimental protocol. For each block one among clean air, happiness body odor or fear body odor and 6 neutral faces were presented. *Note*. Faces are blurred for anonymity purposes.

After the EEG cap and ECG application, participants were seated in a dimly lit, sound-attenuated and electrically shielded room facing an LCD monitor placed ~0.7 m in front of them. ECG electrodes and an olfactometer tube for odor delivery were fitted onto participants. First, there was a 3-min resting-state period. Second, odor pleasantness, intensity, and familiarity were rated before and after the experimental task, with each odor administered individually in a four-second pulse. Participants used on-screen visual-analog scales to provide ratings of pleasantness (from 0 very unpleasant to 100 very pleasant), intensity (0 no odor to 100 very intense odor), and familiarity (0 not familiar at all to 100 extremely familiar) of the odor. Finally, after a brief training phase composed of 6 trials, participants engaged in a passive viewing task, where they were asked to look at neutral faces on the screen while breathing normally. Odors and images were presented in a randomized block design. The odor stimulation session consisted of 21 blocks (7 for happiness odor, 7 for fear odor, 7 for clean air). Each block, lasting around 36 s, presented only one odor and six images. Each image was displayed for 2 s, preceded by a 2-s gray interval. The ISI ranged from 1 to 3 s. Between each block, images were displayed again and after each image participants were asked to rate the valence and arousal of the images using the 9-point Valence and Arousal scales of the Self-Assessment Manikin (SAM) [64]. During the ratings, only clean air was presented. The task lasted around 30 min, while the entire experimental session lasted around 90 min. In Fig. 1 an overview of the study design is presented.

### Heart rate preprocessing

The ECG signal was analyzed offline using MATLAB (MATLAB 2020, version 9.9 R2020b, The MathWorks Inc, Natick, Massachusetts). The continuous signal was segmented into 22 epochs: a 180-second resting-state baseline and 21 epochs corresponding to odor stimulation blocks, each lasting ~36 s. Epochs with irreducible artifacts were excluded from the analysis. The remaining epochs were analyzed using Kubios HRV Analysis Software 3.3.1 (Matlab, Kuopio, Finland). The analysis involved extracting inter-beat (RR) series from the ECG signal using the Pan-Tompkins algorithm [65], artifact removal using a cubic spline interpolation method, and resampling the RR time series at 4 Hz to derive HRV signals. For each epoch, various features were extracted from time (mean HRV, std HRV, RMSSD, pNN50), frequency (LF peak, HF peak, LFnu, HFnu, LF/HF ratio), and nonlinear (SD1, SD2) domains, representing autonomic nervous system (ANS) activity. The baseline estimated during the initial 3-min baseline was then removed for each feature.

### EEG data preprocessing

The EEG signal was analyzed offline using EEGLAB and custom MATLAB scripts. Signals were band-pass filtered between 0.1 Hz and 50 Hz and downsampled to 125 Hz. Bad channels were removed if flat for more than 5 s or poorly correlated with the reconstruction obtained from their adjacent channels. Here we used the default value of *ρ* < 0.8 for the threshold [66]. Removed channels were recovered through spherical spline interpolation. Preprocessed signals were then referenced to the numeric average of all channels. EEG signals were segmented into epochs ranging from −1000 ms to 2000 ms around the visual stimulus, and epochs with abrupt signal changes were discarded after visual inspection. More specifically, epochs containing irreducible and non-stereotyped artifacts (e.g., head movements, electrode movements) were removed as a necessary preprocessing step for the subsequent independent component analysis. Indeed, such non-stereotyped artifacts may quickly introduce a variety of unique scalp patterns into the EEG data, which may in turn confound and compromise ICA decompositions [67]. At the end of this procedure, we retained more than 86% of the epochs. Cleaned epochs were decomposed into sets of statistically independent components through independent component analysis (ICA) [68]. ICLabel was used to tag components as brain, muscle, eye, heart, line noise, channel noise, or other [69] and to reconstruct the signal with only the *brain* components. This procedure allows to clean the EEG signal on the scalp [70] and increases the interpretability of brain activity-related components identifying the brain sources contributing to the signal [71]. In addition, an expert visually inspected and removed potentially misclassified components. Each epoch had a subtractive baseline (estimated in the 1000 ms preceding stimulus administration) removed.

For each subject, grand average ERPs and time-frequency event-related-spectral-perturbations (ERSPs) were estimated for each channel and condition. ERPs were obtained in the −1000 to 2000 ms time range around stimulus administration. ERSPs were obtained in the −500 to 1500 ms time range around stimulus administration. More specifically, time-frequency analysis was performed using a Morlet wavelet transformation on individual trials for each 1-Hz frequency bin between 1 and 30 Hz, using a mother wavelet at 1 Hz with 3-s time resolution (full width at half maximum; FWHM). Time-frequency decompositions were then averaged for each participant and odor condition, and the ERSP was computed as the change in power expressed in decibels (dB) relative to the baseline (−900 to −400 ms) in each frequency bin at each time point. Finally, for visualization purposes, data were grand averaged across each group for each odor condition.

### Statistical analysis

Behavioral data were cleaned and analyzed using the software R [72] and JASP [73]. Odor ratings, recorded before and after the experiment, were analyzed with linear mixed models (LMMs) computed for each dependent variable (pleasantness, familiarity, and intensity) using the *lmer* function (*lme4* package [74]). Models included the interaction between Time (before and after) and Odor (fear, happiness, and clean air), as well as an intercept for the random effect of participants to account for individual variation. Collinearity between predictors was measured by calculating the Variance Inflation Factors (VIF) with the *vif* function (*car* package [75]). All factors showed low collinearity, with values below 2. Normality of the residuals was checked through visual inspection of the q–q plots (quintile-quintile plot), graphical tools in which the quantiles of the data are plotted against the quantiles of a theoretical normal distribution. If the data points fall along a straight line, it suggests that the data are approximately normally distributed. Significant deviations from linearity of observations or non-symmetric scales indicated a deviation from normality of the residuals. In case of significant deviation, values with more than plus or minus 3 Median Absolute Deviation (MAD) from the median were removed. Significant deviations from linearity were found for Intensity odor rating, for which the model was rerun without outliers.

Valence and arousal ratings of the images were analyzed with two 3 × 3 repeated measure analyses of variance (ANOVAs) with Odor as within factor, Group as between factor and Odor × Group interaction as proposed in the pre-registration. Sphericity in the data was corrected with Greenhouse–Geisser correction. The Shapiro-Wilk test was utilised to assess the normality of the distribution. Significant main effects (*p* < 0.05) were followed by Bonferroni correction to control for multiple comparisons. Due to technical problems, for one subject, only 103 data points were available instead of 126.

Each HRV feature was analyzed with a 3 × 3 repeated measure ANOVA with the Odor as a within-subject factor and Group as a between-subject factor, controlling for multiple hypothesis testing with the false-discovery-rate (FDR) correction method [76]. Post hoc analysis was carried out with paired t-tests.

Differences in ERP amplitudes were analyzed for the different regions of interest (ROI) and time windows of interest (WOI). Particularly, we identified 4 spatial clusters of interest, each of which is associated with specific time ranges in which we expected differences in ERP amplitude according to previous literature [77–79] (Table s2). For each ROI, differences in the ERP amplitudes were analyzed for each time point in the relative WOIs. As exploratory analyses, the same ROIs and WOIs were used for analyzing differences in the ERSPs. For this last measure and to consider standard EEG frequency bands (i.e., delta 1–3 Hz, theta 4–7 Hz, alpha 8–12 Hz, beta 13–25 Hz), we considered all the frequencies in the 1–25 Hz frequency range. For each measure and for each cluster of interest, a 3 × 3 repeated measure ANOVA with the Odor (clean air, happiness, fear) as within-subject factor and Group (CONT, DEP, SAD) as between-subject factors was performed. Repeated measure ANOVAs were performed for each time, frequency, and ROI. To further refine the clusters within the ROIs, we controlled multiple hypothesis testing with cluster permutation (*α* = 0.05) [80]. Both ANOVAs and cluster-correction steps were performed using the Factorial Mass Univariate Toolbox (FMUT) [81] and Fieldtrip [80]. Post hoc analyses were carried out by means of paired t-tests for testing within-subject differences (i.e., odor differences for each separate group) and unpaired t-tests for testing between-subject differences (i.e., group differences for each separate odor).

## Results

### Fear and happiness chemosignals were not perceived as different odors

Before and after the EEG experimental task, participants were asked to rate the intensity, pleasantness, and familiarity of the three odor conditions (fear, happiness, and clean air). Participants did not rate the three odor conditions as different in terms of pleasantness (all *β* < 4.05, *t* < 1.61, *p* > 0.11) and familiarity (all *β* < 3.35, *t* < 1.33, *p* > 0.18). The three odors were rated as slightly more intense after the experimental task compared to before (time effect: *β* = 2.64, *t* = 1.95, *p* = 0.052) but there was no difference among odors or Odor × Time interaction (all *β* < 0.83, *t* < 0.44, *p* > 0.64), confirming that the three odor conditions were not consciously perceived as different.

### Fear and happiness chemosignals did not modulate the subjective ratings of the neutral faces

The result of the Shapiro-Wilk test indicates that data on valence rating were normally distributed (*p* = 0.17). For valence ratings, the repeated measure ANOVA did not reveal any significant results (main effect of Odor: F_(1.96, 121.9)_ = 0.55, *p* = 0.57; main effect of Group: F_(2,63)_ = 0.17, *p* = 0.84; interaction F_(3.87, 121.9)_ = 0.90, *p* = 0.46). The result of the Shapiro-Wilk test indicates that data on arousal ratings were not normally distributed (*p* = 0.025). For arousal ratings, the mixed ANOVA showed a main effect of Group (F_(2,63)_ = 4.54, *p* = 0.014), in which the SAD group rated the images as more arousing than the CONT group (*p* = 0.02) independently of the odor condition. No differences were found between CONT and DEP groups (*p* = 0.07) or between SAD and DEP groups (*p* = 1.0). No other significant results were found (main effect of Odor: F_(1.69, 106.5)_ = 0.11, *p* = 0.35; interaction F_(3.38, 106.5)_ = 0.79, *p* = 0.51).

### Fear and happiness body odors increased HRV

**Fig. 2:**
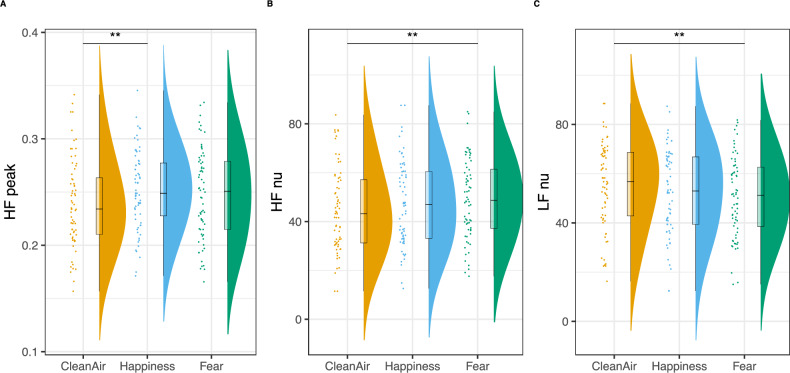
HRV odor main effect. Raincloud plots representing HF peak (**A**), HF power (**B**), and LF power (**C**) values across the three odor conditions. Clouds represent distribution, boxplots depict the median (horizontal black line) and quartile ranges of the distribution, whiskers indicate maximum and minimum values, colored dots represent individual values. **p < 0.01.

The HRV analyses showed a significant main effect of Odor on the peak frequency of the high-frequency band (HF peak, F_(2,130)_ = 5.85, *p* = 0.004), the normalized high-frequency band (HF nu, F_(2,130)_ = 6.14, *p* = 0.003), and the normalized low-frequency band (LF nu, F_(2,130)_ = 6.15, *p* = 0.003). Post hoc analysis showed that the HF nu increased during fear odor exposure, while LF nu decreased, compared to the clean air condition, as they are two equivalent measures (i.e., LF nu = 1-HF nu). Finally, the HF peak was higher in the happiness odor compared to the clean air condition (Fig. 2).

### Emotional BOs affect the processing of neutral faces regardless of the presence of social anxiety or depressive symptoms

**Fig. 3:**
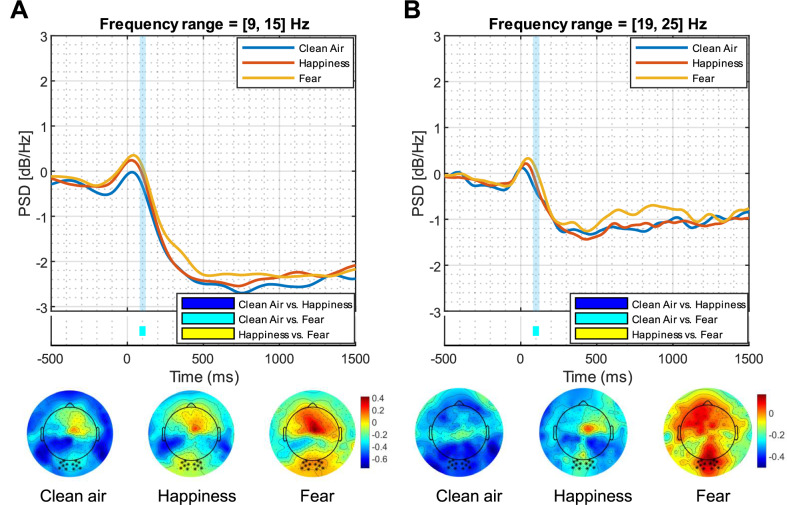
ERSP responses to the three odor conditions in the occipital cluster. *Top panels*. Time course of grand-average event-related power spectral density in the 9–15 Hz (**A**) and in the 19–25 Hz (**B**) frequency ranges over the significant electrodes for clean air (blue line), happiness body odor (red line), and fear body odor (yellow line) conditions. The light blue area represents the significant time window (80–120 ms). *Bottom panels.* Topography of the mean event-related power averaged over the significant time points and frequency bands for clean air, happiness body odor, and fear body odor conditions.

**Fig. 4:**
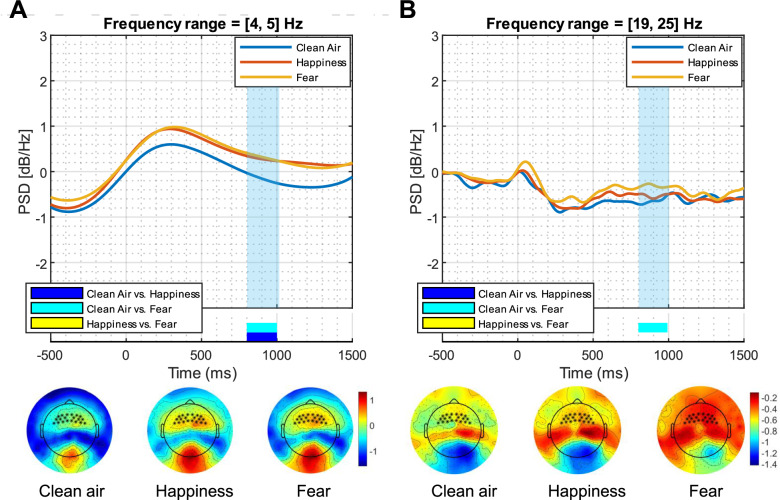
ERSP responses to the three odor conditions in the fronto-central cluster. *Top panels.* Time course of grand-average event-related power spectral density in the 4–5 Hz (**A**) and in the 19–25 Hz (**B**) frequency ranges over the significant electrodes for clean air (blue line), happiness body odor (red line), and fear body odor (yellow line) conditions. The light blue area represents the significant time window (800–1000 ms). *Bottom panels.* Topography of the mean event-related power averaged over the significant time points and frequency bands for clean air, happiness body odor, and fear body odor conditions.

For ERPs data, repeated measure ANOVA analyses did not show any significant effect for Odor, Group, and Odor × Group interaction after correcting for multiple hypothesis testing. The same was used to analyze ERSPs measures. A repeated measure ANOVA with Odor as within-subject factor, Group as between-subject factor and their interaction was employed. The mixed ANOVAs revealed two Odor main effects in the time window 80–120 ms in the occipital cluster for the frequency range 9–15 Hz and 19–25 Hz, as reported in Fig. 3A, B. Specifically, in both clusters a higher power for the fear condition emerged compared to the clean air condition. In addition, the repeated measure ANOVAs yielded two Odor main effects in the time window 800–1000 ms in the fronto-central cluster for the frequency range 4–5 Hz and 19–25 Hz, as reported in Fig. 4A, B. In the first cluster (4–5 Hz), both happiness and fear conditions resulted in increased power compared to the clean air condition. In the second cluster (19–25 Hz), only the fear condition resulted in higher power compared to the clean air condition.

## Discussion

The current study investigated how neutral facial expressions are subjectively perceived and processed when presented alongside happiness and fear chemosignals, particularly in individuals with depressive and social anxiety symptoms. The study employed a comprehensive approach, investigating the subjective evaluation, the autonomic responses, and the neural processing of the visual stimuli in individuals with depressive symptoms, individuals with social anxiety symptoms, and healthy controls. Our hypothesis that happiness and fear chemosignals modulate the subjective and psychophysiological processing of neutral faces differently in individuals with depressive symptoms, individuals with social anxiety symptoms and healthy controls was only partially supported.

With respect to the subjective ratings, the group with social anxiety symptoms evaluated the neutral faces as more arousing compared to the control group, regardless of the odor condition, in line with previous studies reporting that ambiguous or neutral faces are far from being neutral for individuals with affective disorders [82, 83]. Apparently, this biased evaluation has a more powerful influence on the subjective ratings compared to the contextual emotional chemosignals.

With respect to the psychophysiological measures, results showed no significant differences between the groups of participants, in contrast with our hypotheses, but they support the modulatory effect of the emotional chemosignals on the autonomic and neural responses. Both happiness and fear chemosignals, during passive viewing of neutral faces, were associated with increased HRV compared to the clean air condition. According to the neurovisceral integration model [84], HRV, particularly high-frequency HRV, reflects the effective functioning of neural networks involved in the interplay between emotions and cognitive processes. In this study, the heightened task-related HRV in response to faces coupled with emotional chemosignals suggests enhanced adaptability in managing autonomic reactions and focused attention [85–87]. This adaptability facilitates improved emotion regulation and greater autonomic flexibility, enhancing the individuals’ ability to effectively respond to neutral visual stimuli influenced by emotional chemosignals.

From a neural perspective, the EEG time-frequency analyses provided evidence of a modulatory effect of emotional chemosignals on delta and beta oscillations. First, increased event-related delta oscillations were observed in response to neutral faces presented with both emotional chemosignals compared to clean air. Past research has associated heightened delta power with emotionally relevant stimuli [21, 22, 88–91], indicating its role in motivational processes tied to the brain’s reward system [21, 91]. This study extends existing literature by demonstrating, for the first time, that happiness and fear chemosignals influence the neural processing of otherwise neutral stimuli. This modulation results in heightened motivated attention and affective dispositions toward the emotional content of the stimuli. Second, the fear chemosignal led to a reduced beta desynchronization compared to the clean air condition in both early (~80–120 ms) and late (~800–1000 ms) stages of stimulus processing, respectively in posterior and anterior sites. Notably, a significant difference between clean air and fear chemosignal emerged in both early and late stages. However, no difference between the two chemosignal conditions, or between the happiness chemosignal and clean air, was reported. This reduction in beta desynchronization aligns with previous research indicating that beta oscillations are involved in cognitive processes, with decreased beta activity occurring when a novel event disrupts the current cognitive state [92, 93]. Beta oscillations seem to be also content-specific: higher beta desynchronization is observed when processing high-arousal pictures compared to low-arousal pictures [94], suggesting that they reflect both affective and cognitive processes. In the current study, the fear chemosignal was presented before the neutral faces, potentially necessitating preferential processing and utilizing neural resources that might otherwise be available for processing the faces. This scenario could explain the observed reduced beta desynchronization in response to neutral faces presented with the fear chemosignal, consistent with a previous study using anxiety and neutral chemosignals [56]. The unique impact of the fear chemosignal on beta activity might be attributed to its well-established role as a warning signal, increasing vigilance and attention to the surroundings that predispose the organism to handle potentially dangerous situations [95], demanding more neuronal resources. In contrast, the processing of the happiness chemosignal, lacking direct consequences for species survival [96], may be less automatic or prioritized.

It is important to emphasize that the current findings cannot be attributed to a perceptual distinction between the three odor conditions. Indeed, consistent with earlier studies, the emotional body odors received similar ratings in terms of intensity, pleasantness, and familiarity when compared to the clean air condition [43, 45]. Consequently, the observed effects occurred independently of conscious awareness of the odor stimuli, affirming their significance in social communication, even when they are perceived subliminally, akin to real-life situations.

It is worth noting that the effects of the contextual emotional chemosignals emerged through the event-related time-frequency EEG analysis, but not using the standard ERP analysis. This may simply be due to lack of power to detect ERP differences, but also to the methodology used. Indeed, the time-frequency approach provides several methodological benefits in contrast to conventional ERPs in studying emotional processing, distinguishing between affective disposition and top-down processing [97]. In addition, event-related oscillations encompass not just stimulus-driven oscillations akin to ERPs but also include induced oscillations that are not synchronized with the event phase of the stimulus, providing valuable information that is not captured by ERPs alone [98]. Contrary to our hypotheses, no significant differences between groups were found. On one hand, this null finding may be due to the methodological approaches. Specifically, while the use of ambiguous stimuli (i.e., the neutral facial expression) allowed the chemosignals to modulate their perceived valence and arousal, this was probably not strong enough to reveal group differences. Moreover, the study focused on subclinical forms of affective disorders in a healthy young population, potentially limiting its ability to uncover small effects. This approach allows a better understanding of the initial mechanisms related to the social impairment, offering insight into future indications of vulnerability, while mitigating the influence of confounding factors such as medication or the chronic nature of the disorders. However, individuals with subclinical forms of affective disorders may present varying levels of symptomatology that could dilute the observed effects. On the contrary, clinical forms of affective disorders involve more severe and pervasive symptoms compared to subclinical forms, exhibiting more pronounced impairment in various domains, making these effects easier to detect. On the other hand, the evidence that the perception of neutral stimuli presented in the context of emotional chemosignals appears to be unaffected by the altered social skills suggests that emotional chemosignals may be a potential therapy support for future treatments. Future research should explore deeper and confirm this result in clinical populations and treatment settings. The findings of this study carry implications not only for therapeutic interventions but also for real-world contexts. Indeed, in daily encounters, individuals often confront ambiguous stimuli, and body odors serve as a significant factor in interpreting situational meaning. However, to effectively translate these findings into everyday scenarios, it is imperative to develop more ecologically valid methodologies, encompassing the collection and presentation of body odors. In this regard, the integration of virtual reality presents a promising avenue for future research.

When interpreting our findings, it is important to acknowledge some limitations. First, the sample size was relatively small as we failed to reach the targeted sample size due to technical issues. For this reason, present results should be taken with caution. Second, the sample included only women, thereby limiting the ability to extend our findings to the male population. The decision to include only women was made in order to reduce gender-related influences. Indeed, prior research demonstrated that women typically exhibit heightened olfactory abilities [99] and greater awareness of odors [100] compared to men. Third, the use of clean air as a control condition did not allow us to conclude that the effect we observed is specific to the emotion conveyed by the body odor and not a general effect of social presence. Future research should incorporate a neutral body odor condition to explore whether the outcomes observed in the current study stemmed from the social context conveyed by body odors or from the particular emotion each odor was intended to convey.

Taken all together, the results of this study are the first attempt to simultaneously investigate the subjective, autonomic, and neural responses toward neutral facial expressions presented in the context of emotional chemosignals in individuals with depressive symptoms, social anxiety symptoms and healthy controls. Both happiness and fear chemosignals acted in a similar vein in enhancing the vagal tone of the participants and in modulating the neural processing of ambiguous social visual stimuli. The present study provides novel evidence that human emotional chemosignals are powerful contextual cues and it constitutes a significant step forward in understanding the social role that emotional chemosignals play in both healthy populations and in populations with affective disorders.

### Supplementary information

Supplementary material

## Data Availability

The data that support the findings of this study have been deposited in the Open Science Framework (https://osf.io/3nb4a/).

## Code availability

The codes that support the findings of this study have been deposited in the Open Science Framework (https://osf.io/3nb4a/).
