# Supplementary material for: Emotion perception through the nose: how olfactory emotional cues modulate the perception of neutral facial expressions in affective disorders
Source: Transl Psychiatry. 2024 Aug 24;14:342. doi: 10.1038/s41398-024-03038-z (PMC11344772; doi:10.1038/s41398-024-03038-z)
Supplement: Supplementary file 1 — Supplementary material [file 41398_2024_3038_MOESM1_ESM.docx]

**Supplementary material**

*Sniffin’ Sticks test*

Olfactory function was assessed with the computer-testing version of the standardized clinically approved “Sniffin’ Sticks” test (Burghart Instruments, Wedel, Germany^1^). Due to the SARS-CoV-2 pandemic and to avoid the spread of the disease, the procedure slightly changed from the original version, and a single-use smell test system was adopted. Each odor was presented on a single-use paper strip, which was then handed over to the participant. The participant smelled the paper and answered the question. The paper strip was then discarded, and the procedure was repeated for the next stick^2–4^. Three different olfactory functions were assessed. First, odor identification was measured by presenting 16 common odors, each presented with four verbal descriptors in a multiple forced-choice format (three distractors and one target). Second, odor discrimination was assessed over 16 trials using a 3 Alternative Force Choice task (3AFC). For each discrimination step, three pens were presented in random order, two containing the same odor and the third containing the target odor. Third, the odor detection threshold was determined for n-butanol with 16 stepwise dilutions using an ascending limits procedure^2,5^ based on a three-alternative forced choice task (3AFC). Only participants obtaining a TDI score in the normosmic range (i.e., above or equal to 30.5) were included in the study.

*Body odor collection and preparation*

Body odor samples were collected at the Instituto Superior de Psicologia Aplicada (Lisbon, Portugal) before the beginning of the experimental session from a different group of participants [called “body odor donors”, 32 healthy Caucasian participants (16 females) aged between 18 and 35 (mean = 21.64, SD = 3.63)]. Body odor donors reported to be heterosexual, nonsmokers, not to have past or current psychological disorders and to not take medications. Body odors were collected through sterilized cotton pads attached to the armpit zone while donors were exposed to videos that induced emotional states of fear or happiness. The videos were composed of several short video clips (already used in previous studies to induce fear and happiness; ^6–8^) with a total average duration of 25 min. Fear and happiness body odor collections were separated by a week’s interval, with participants following a strict behavioral and dietary regimen every 2 days prior to each odor collection session to avoid sweat contamination (see ^9^ for a detailed list of restrictions). Before and after each emotional state induction, donors rated to what extent they felt angry, fearful, happy, sad, disgusted, neutral, surprised, calm, and amused on separate 100-point Likert scales that ranged from 1, not at all, to 100, very much.

To analyze the rating of the emotional states, completed before and after the body odor collection, we performed a linear mixed model with the interaction between the Emotion (fear, anger, disgust, surprise, joy, sadness, funny, neutral and calm), the Odor collection (happiness and fear body odors) and the Time (before and after emotional state induction) using *lmer* function (*stats* package^10^) and then explored them using the *anova* function of the same package. The participant’s ID was included as random factor. Significant effects (p < .05) were followed by Tukey’s test to control for multiple comparisons.

The rating of the emotional state performed before and after each emotional state induction confirmed that the emotional induction procedure worked. Indeed, the ANOVA yielded a significant main effect of the Emotion (F_(8,1050)_ = 97.45, p < .001), a significant Time × Odor interaction (F_(1,1050)_ = 6.99, p = .008), Time × Emotion (F_(8,1050)_ = 23.56, p < .001), Odor × Emotion (F_(8,1050)_ = 23.18, p < .001), Time × Odor × Emotion (F_(8,1050)_ = 27.53, p < .001). The Tukey post-hoc test revealed that the donors after the fear induction, compared to before, felt more fear (p < .001), more disgust (p = .028), more surprise (p < .001), less joy (p < .001), less fun (p < .001), less neutral (p < .001) and less calm (p < .001), while they did not differ for the level of anger (p = .126) and sadness (p = .962). After the happiness induction, donors reported to feel more joy (p = .001), more fun (p < .001), less neutral (p = .007), while they did not differ for the level of fear (p = .473), anger (p = .999), disgust (p = .999), surprise (p = .058), sadness (p = .934), calm (p = .782). See Table s1 for mean and SD of emotional state ratings.

After emotional state induction, pads were removed, frozen individually in amber vials at −80 °C, and then shipped in dry ice to the University of Padova (Italy), where they have been stored again in a −80 °C freezer.

As in previous studies^6,9^, in order to reduce the effects of interindividual variability in sweat production, body odor pads were prepared for presentation as follows: while still frozen, each pad obtained from the sweat donors’ armpits was cut into eight equal parts. Using a randomization script, pad pieces from four sweat donors (two females, two males; two from the left and two from the right armpits) of the same emotional states - fear or happiness - were combined to create a super-donor.

# Table s1

|  | **Happiness induction** | | **Fear induction** | |
| --- | --- | --- | --- | --- |
| **Emotional state** | **Pre** | **Post** | **Pre** | **Post** |
| Fear | 8.16 (13.48) | 1.64 (4.98) | 8.81 (15.38) | 54.06 (30.53) |
| Anger | 1.64 (3.38) | 1.19 (2.81) | 2.03 (3.33) | 11.93 (16.51) |
| Disgust | 0.67 (1.87) | 0.93 (2.51) | 0.68 (1.70) | 13.26 (16.79) |
| Surprised | 9.90 (18.79) | 21.26 (22.47) | 8.74 (12.97) | 36.64 (25.29) |
| Joy | 42.10 (21.65) | 58.71 (18.43) | 44.77 (24.03) | 16.58 (20.59) |
| Sadness | 6.22 (11.12) | 3.55 (7.65) | 7.35 (11.89) | 9.55 (13.97) |
| Funny | 26.97 (21.65) | 56.02 (20.30) | 33.87 (24.88) | 14.84 (20.16) |
| Neutral | 47.22 (24.99) | 32.68 (23.03) | 44.68 (28.17) | 15.12 (18.43) |
| Calm | 54.22 (23.90) | 49.97 (29.59) | 56.29 (24.75) | 13.35 (14.92) |

**Table s2. Regions of interest (ROI) and windows of interest (WOI) selected for the ERPs analyses.**

| **Cluster** | **Electrodes** | **Time range** |
| --- | --- | --- |
| Occipital (O) | 124 123 136 135 134 146 149 148 158 157 166 156 | P100 (80 - 120) ms |
| Parietal (P) | 105 106 107 113 114 115 121 122 159 167 168 169 175 176 177 | N170 (150 - 180) ms |
| Centro-Parietal(CP) | 45 53 60 66 80 79 78 89 88 100 144 155 164 131 143 154 130 142 129 | LPP1(400 - 600) ms  LPP2(800 - 1000) ms  LPP3(1200 - 1500) ms  LPP4(1600 - 1800) ms |
| Fronto-Central (FC) | 50 41 42 36 30 24 29 23 16 22 14 5 6 7 224 215 207 214 206 205 | LPP1(400 - 600) ms  LPP2(800 - 1000) ms  LPP3(1200 - 1500) ms  LPP4(1600 - 1800) ms |

**References**

1 Hummel T, Sekinger B, Wolf SR, Pauli E, Kobal G. ‘Sniffin’ sticks’: olfactory performance assessed by the combined testing of odor identification, odor discrimination and olfactory threshold. *Chem Senses* 1997; **22**: 39–52.

2 Besser G, Liu DT, Renner B, Mueller CA. Self-administered testing of odor threshold and discrimination using sniffin’sticks—reviving the “odor-curves-on-paper” method. *Chemosens Percept* 2020; **13**: 71–77.

3 Mueller CA, Grassinger E, Naka A, Temmel AFP, Hummel T, Kobal G. A self-administered odor identification test procedure using the “sniffin’sticks”. *Chem Senses* 2006; **31**: 595–598.

4 Wirkner K, Hinz A, Loeffler M, Engel C. Sniffin’Sticks Screening 12 test: Presentation of odours on filter paper improves the recognition rate. *Rhinology* 2021.

5 Pössel M, Freiherr J, Horstmann A. Rapid assessment of olfactory sensitivity using the “Sniffin’Sticks”. *Chemosens Percept* 2020; **13**: 37–44.

6 de Groot JHB, Smeets MAMAM, Rowson MMJMJ, Bulsing PJ, Blonk CG, Wilkinson JE *et al.* A sniff of happiness. *Psychol Sci* 2015; **26**: 684–700.

7 de Groot JHB, Semin GR, Smeets MAM. Chemical communication of fear: A case of male–female asymmetry. *J Exp Psychol Gen* 2014; **143**: 1515.

8 De Groot JHB, Semin GR, Smeets MAM. I can see, hear, and smell your fear: Comparing olfactory and audiovisual media in fear communication. *J Exp Psychol Gen* 2014; **143**: 825.

9 de Groot JHB, Smeets MAM, Kaldewaij A, Duijndam MJA, Semin GR. Chemosignals communicate human emotions. *Psychol Sci* 2012; **23**: 1417–1424.

10 Team RC. R: A language and environment for statistical computing (3.4. 3). 3.4. 3. 2017.
